# Scorpion stings in pregnancy: an analysis of outcomes in 66 envenomed pregnant patients in Iran

**DOI:** 10.1590/1678-9199-JVATITD-2019-0039

**Published:** 2020-04-30

**Authors:** Mahin Najafian, Ahmad Ghorbani, Mahvash Zargar, Masoumeh Baradaran, Nafiseh Baradaran

**Affiliations:** 1 Department of Obstetrics and Gynecology, School of Medicine, Ahvaz Jundishapur University of Medical Sciences, Ahvaz, Iran.; 2 Toxicology Research Center, Ahvaz Jundishapur University of Medical Sciences, Ahvaz, Iran.

**Keywords:** Scorpionism, Pregnancy complications, Stillbirth, Preterm birth

## Abstract

**Background:**

Scorpionism is one of the most important health problems in tropical regions, which unfortunately results in thousands of deaths annually. Pregnant women are potential victims in areas with high scorpion-sting prevalence. Limited medical data are available on the effects of scorpion envenomation in pregnant women. This study aimed to examine the effect of scorpion envenomation on pregnancy outcomes in 66 cases.

**Methods:**

The present descriptive/analytical cross-sectional study was performed on 66 scorpion-envenomed pregnant women referred to the clinical toxicology unit of Ahvaz Razi Hospital in Iran during 2015-2017. The variables assessed in all cases, via questionnaire and hospital medical records, were: age, patient residency, gestational week, status of the fetus, laboratory anomalies, clinical severity of envenomation, sting site and scorpion species. Pregnancy outcome (miscarriage, stillbirth, preterm birth, normal delivery) and status of the newborns were also evaluated. Data were analyzed using SPSS ^®^ software (version 24.0).

**Results:**

The following pregnancy outcomes were recorded from envenomed pregnant women: miscarriage = 1.5% (n = 1), stillbirth = 4.5% (n = 3), preterm birth = 10.6% (n = 7), normal birth = 83% (n = 55). Among participants whose pregnancy led to birth, 11(17.7%) cases had prenatal-neonatal complications. Neonatal complications, including Apgar score less than 8 points at 5 min, were found in 7 (11.3%) preterm birth cases and in 4 (6.4%) normal birth cases, along with birth weight below 2500 g in normal births. A significant relationship was found between adverse pregnancy outcomes and bite location, as well as scorpion species, but no relationship was found with other variables.

**Conclusion:**

Envenomation significantly contributes to preterm birth. Moreover, the location of bites and the type of scorpion species have a decisive role in the pregnancy outcome of scorpion-envenomed pregnant women.

## Visual Abstract

**Figure:**
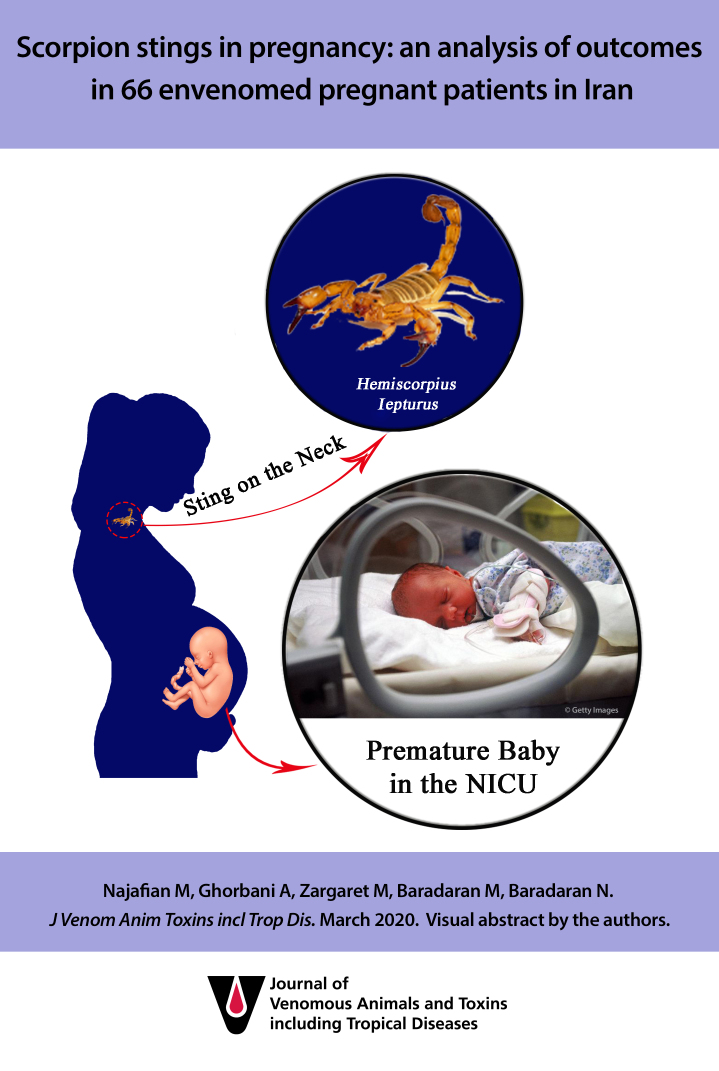
Scorpion stings in pregnancy: an analysis of outcomes in 66 envenomed pregnant patients in Iran

## Background

Scorpion stings have been reported in most regions of the world. Geographically, scorpions are found at latitudes between 50 degrees north and 52 degrees south of the equator) [1]. Scorpion envenomation is one of the most important health problems in the tropical regions, which unfortunately results in thousands of deaths, annually [1-4]. Scorpion stings constitute a major health problem in Iran (45000-50000 cases - about 19 death per year) and adjacent countries (Iraq, Pakistan, Saudi Arabia, Oman, Yemen, and the United Arab Emirates). Scorpion stings are more prevalent in the southern and southwestern regions of Iran including Khuzestan province, where their high frequency has caused severe clinical manifestations [5, 6]. According to the World Health Organization, despite the large number of scorpion envenomings, the actual incidence rates for scorpion stings in different geographical regions and countries are not clear [7].

Scorpions are venomous arthropods, members of the class *Arachnida*and order*Scorpiones* [8]. So far, about 2200 scorpion species have been identified and introduced [9], of which only about 25 are considered life-threatening to humans [10]. *Hemiscorpius lepturus, Androctonus crassicauda*, and*Mesobuthus eupeus*are the main species responsible for stings in Iran. The species *H. lepturus* is endemic in areas of southwestern Iran, where *Androctonus crassicauda* is the second most common species [11-13]. 

Scorpion envenomation mostly causes local and systemic manifestation. Localized pain is the first symptom in most cases. The severity of symptoms depends on the scorpion's species, age and size. Children are more likely to suffer from severe envenomation [14, 15]. The local signs of scorpion sting include itching, erythema, local swelling, and ascending hyperesthesia that persists for several weeks [16]. 

Systemic manifestations are particularly prominent due to different scorpion venom toxins that affect sodium channels, block potassium and calcium channels, or modify chloride channels [17-20]. Thus, scorpion envenomation can be classified into three categories based on the intensity of the initial symptoms: mild, moderate or severe (Table 1). 

**Table 1.** Classification of scorpion envenomation based on the intensity of the initial symptoms [16-20]

Scorpion envenomation	Symptoms
Mild	Pain, edema, erythema, and sweating symptoms are present.
Moderate	Nausea, abdominal pain, tachypnea, tachycardia or bradycardia, mild hypertension, sweating, high fever, restlessness, hypersalivation, priapism, and hyperglycemia symptoms are evident.
Severe	Cardiovascular complications (congestive heart failure, hypertension and cardiac arrhythmias), pulmonary edema (edema* *and* *respiratory distress syndrome), gastrointestinal problems (acute pancreatitis*)*, metabolic complications [hyperglycemia, hyperkalemia, hypokalemia or imbalance of the acidic and basic (alkaline) compounds in blood], and neurological symptoms (hypertensive encephalopathy, coma, or seizure).

Scorpions have been observed in many habitats and can survive under severe conditions. Some scorpion species have adapted their activity inside or around human residential areas, thereby increasing the probability of their encounters with humans. So, in these cases, scorpions threaten more persons [21]. Considering the high scorpion prevalence in some areas, pregnant women and mothers may be potential victims of scorpion envenomation. Some previous studies in Khuzestan province in Iran have reported that most scorpion-sting victims were female homemakers [11, 22]. However, the available medical data on the effects of the scorpion envenomation on pregnancy outcomes are not only insufficient but also somewhat controversial [23]. The aim of the present study was to examine the effect of scorpion envenomation on pregnancy outcomes, maternal and prenatal-neonatal complications, in pregnant women referred to the Clinical Toxicology Unit of Ahvaz Razi Hospital (Khuzestan province, southwestern Iran).

## Methods

This descriptive/analytical cross-sectional study was conducted retrospectively in the Clinical Toxicology Unit of Ahvaz Razi Hospital (Khuzestan province, Iran), from October 1, 2015 to October 1, 2017. After obtaining ethics approvals from the Research Ethics Committee (approval number: IR.AJUMS.REC.1397.547 in Nov. 3, 2018) and written informed consent, women who had been envenomed by a scorpion and referred to Ahvaz Razi Hospital were selected for the study. Medical records including maternal age, gestational age, scorpion species, patient residency, laboratory disorders, sting site, sting severity, and sting time were collected using both medical files and a questionnaire administered during the hospital visit. Pregnancy and neonatal outcomes were evaluated from hospital medical records. Participants lacking either accurate information about the treatment process or complete medical records were excluded from these analyses. Finally, a total of 66 pregnant scorpion-envenomed women were selected.

The envenomed women were from Ahvaz and such surrounding cities as Molasani, Kut Abdollah, Hamidieh and Shadegan. Some brought the scorpion that stung them. The scorpion species were determined by conforming to Farzanpay’s key of identification [24]. The scorpion that stung the remaining referents was denominated an unidentified scorpion.

Scorpion envenomation was classified into three categories based on the intensity of the initial symptoms: mild, moderate or severe [16-20]. Treatment was divided into three categories: supportive (antihistamines, analgesics, antibiotics, steroids, anti-tetanus, etc.), specific (antivenom), and advanced (based on the involved organ including dialysis in kidney failure).

Pregnancy outcomes include normal birth, miscarriage, stillbirth and preterm birth. Miscarriage, stillbirth, and preterm birth were classified as adverse pregnancy outcomes. 

 The “Apgar Score” method was used for evaluation of the newborn infants. Scores of 8 and above were considered generally normal [25]. Malformation of neonates was evaluated based on clinical examination by a pediatrician.

Finally, statistical relationships were determined between adverse pregnancy outcomes and the following variables: maternal age, scorpion species, patient location, laboratory anomalies, gestational age, bite location, severity of scorpion sting, fetal/ neonatal complications and maternal complications (such as hypertension, chest pain and maternal bleeding).

The data were compiled using Microsoft Excel ^®^ and analyzed using the software SPSS ^®^ (version 24.0). The *mean*±*SD* of the variables was calculated. Differences between groups were analyzed using a *t*-test for independent samples. To verify possible differences between nominal scaled variables, a chi*-*square test was performed. Significance was accepted at the P < 0.05 level.

Reliability reported for the data of this study was measured using test-retest with ICC values ranging between 0.91 and 0.94. Validity was analyzed using the PEDro scale [26] by two independent reviewers, with disagreements resolved via consensus.

## Results

A total of 66 pregnant women with scorpion envenomation were studied. The mean age of the participants was 26.8 years (range: 17-42 years). The minimum gestational age was 5 weeks and the maximum was 40 weeks. The mean gestational age was 24.27 (M = 24.27, SD = 2.2 weeks). All of the women received antivenom 6 to 12 hours after the envenomation.

**Figure 1.**
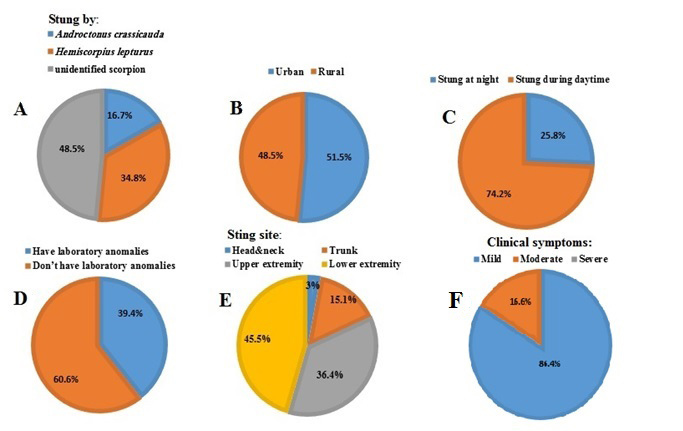
Frequency percentage of different variables of envenomed pregnant women referred to Ahvaz Razi Hospital, Iran.

Of the total pregnant women, 23 cases (34.8%) were envenomed by *H. lepturus*, 11 cases (16.7%) by *A. crassicauda*, and 32 cases (48.5%) were envenomed by unidentified scorpion species (Figure 1, A). *H. lepturus* was distinguished by morphological characteristics including transparency to turbid yellow body color, pedipalps and legs lighter in color, reddish brown pedipalps and brown spots at the end of the legs. The moving fingers of chelicerae have two branches. The following three main features were considered for recognition of *A. crassicauda*: body color ranging from dark brown to black, narrower pedipalp (Chelae) than patella in adults, and pedipalp fingers with many denticles.

 Thirty-four cases (51.5%) of stung pregnant women were residing in urban areas and 32 cases (48.5%) were rural residents (Figure 1, B). Furthermore, 17 cases (25.8%) were stung at night versus 49 cases (74.2%) during the daytime (Figure 1, C). In 26 cases (39.4%) laboratory anomalies were evident and no laboratory anomalies was observed among the remaining participants (n = 40, 60.6%) (Figure 1, D). Sting sites were head and neck (n = 2, 3%), trunk (n = 10, 15.1%), upper extremity (n = 24, 36.4%), and lower extremity (n = 30, 45.5%) (Figure 1, E). The most common sting site was a lower extremity. Fifty-seven cases (86.4%) had mild clinical symptoms, and 9 (16.6%) cases showed moderate clinical symptoms of scorpion envenomation. None of the patients presented severe clinical symptoms (Figure 1, F)*.*

**Figure 2.**
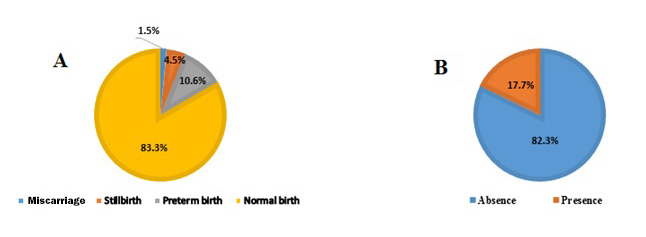
Frequency percentage of **(A)** pregnancy outcomes, and **(B)** prenatal-neonatal complications, in envenomed pregnant women.

The following results are related to pregnancy outcome: miscarriage = 1.5% (n = 1), stillbirth = 4.5% (n = 3), preterm birth = 10.6% (n = 7), normal birth = 83% (n = 55) (Figure 2, A). Overall, 16.6% of pregnancy outcomes were adverse. Miscarriage occurred at the gestational age of 10 weeks. Preterm birth at gestational ages of 26, 28, 30, 32, and 34 weeks happened 2, 2, 1, 1, and 1 case, respectively. Stillbirth occurred at gestational ages of 22, 24, and 30 weeks. All of the preterm births, except two (happened in third and fourth week) happened within one to two weeks after envenomation.

Among 62 scorpion-envenomed women (miscarriage and stillbirth were excluded) whose pregnancy led to birth 11(17.7%) cases had prenatal-neonatal complications, but no prenatal-neonatal complications were recorded among the rest of the participants (n = 51, 82.3%) (Figure 2, B). Neonatal complications including prematurity and Apgar score less than 8 points at 5 min was found in 7 (11.3%) preterm birth cases. However, neonatal complications following normal birth were also found in 4 (6.4%) cases that included Apgar score less than 8 points and birth weight less than 2500g (Table 2).

**Table 2.** Prenatal-neonatal complications in stung women

Prenatal-neonatal complications	Numbers	Type
Following preterm birth	7 (11.3%)	Apgar score less than 8 points at 5 min
Following normal birth	4 (6.4%)	Apgar score less than 8 points at 5 min
		Low-birth-weight neonates (under 2500g)

Tables 3-7 display the frequency of pregnancy outcomes based on the variables maternal age, gestational age, scorpion species, patient residency, laboratory disorders, fetal/neonatal complications, sting site, severity of scorpion sting and sting time. No maternal complications were observed.

**Table 3.** Frequency of pregnancy outcomes based on maternal age and gestational age in scorpion-envenomed pregnant women.

Pregnancy outcome	Numbers (%)	Maternal age (Mean)	Gestational age (Mean)
**Adverse**	11	25.2727	24.3273
**Normal**	55	28.0364	26.7273

**Table 4.** Frequency of pregnancy outcomes based on scorpion species.

Stung by scorpions	Pregnancy outcome		Total
	Adverse	Normal	
*A. crassicauda*	0	11	11
	(0%)	(100%)	
*H. lepturus*	4	19	23
	(17.4%)	(82.6%)	
Unknown species	7	25	32
	(21.9%)	(78.1%)	
Total	11	55	66
	(16.7%)	(83.3%)	

**Table 5.** Frequency of pregnancy outcomes based on patient residency, sting time, laboratory anomalies and prenatal-neonatal complications.

Pregnancy outcome	Residential areas		Sting time		Laboratory anomalies		Prenatal-neonatal complications	
	Rural areas	Urban areas	Night	During day time	Have	Don’t have	Present	Absence
Adverse	7	4	4	7	3	8	7	0
	(20.6%)	(12.5%)	(23.5%)	(14.3%)	(11.5%)	(20%)	(100%)	
Normal	27	28	13	42	23	32	4(7.3%)	51(92.7%)
	(79.4%)	(87.5%)	(76.5%)	(85.7%)	(88.5%)	(80%)		
Total	34	32	17	49	17	49	11	51

**Table 6.** Frequency of pregnancy outcomes based on sting location.

Sting location	Pregnancy outcome		Total
	Adverse	Normal	
Head, neck and trunk	6	6	12
Extremity (upper and lower)	5	49	54
Total	11	55	66

**Table 7.** Frequency of pregnancy outcomes based on severity of scorpion sting.

Severity of scorpion sting	Pregnancy outcome		Total
	Adverse	Normal	
Mild	10	47	57
	(17.5%)	(82.5%)	
Moderate	1	8	9
	(11/1%)	(88.9%)	
Severe	0	0	9
	(0%)	(0%)	
Total	11	55	66

All adverse pregnancy outcomes were associated with prenatal-neonatal complications. The relationship between adverse pregnancy outcomes and various variables was assessed by *t-test* and *chi-square* test. The results with obtained P-values are displayed in Table 8.

A future study should investigate the relationship between adverse pregnancy outcomes and scorpion species, given that unknown scorpions (Table 4) were not included in the statistical analysis, and the relationship was assessed between known species and adverse pregnancy outcomes.

**Table 8.** Relationship between adverse pregnancy outcomes and eight variables

Variable	Relationship with adverse pregnancy outcomes		Statistical significance
	P-value in t-test	P-value in chi-square	
Maternal age	0.16	0.16	No
Scorpion species		0.043	Yes
Patient residency		0.27	No
Sting time		0.37	No
Laboratory anomalies		0.36	No
Gestational age	0.38		No
Sting location		0.02	Yes
Severity of scorpion sting		0.43	No

A statistically significant difference was found between adverse pregnancy outcomes and the scorpion species, as well as the envenomation site (p < 0.05). 

## Discussion

Despite its importance, there is insufficient information on the effects of scorpion envenomation on pregnancy and neonatal outcomes, and unfortunately the available data is controversial [23]. Therefore, the present study aimed to assess the possible toxic effects of scorpionism on pregnancy and neonatal outcomes. The present study was conducted on 66 scorpion-envenomed pregnant women referred to the clinical toxicology unit of Ahvaz Razi Hospital. Statistical analysis of data obtained from the medical records revealed a significant association between adverse pregnancy outcomes (miscarriage, stillbirth, and preterm birth) and scorpion sting. In addition, a statistically significant relationship was determined between adverse pregnancy outcomes and the scorpion species, as well as the envenomation site, but there was no relationship between adverse pregnancy outcomes and other variables including: maternal age, laboratory anomalies, gestational age, fetal/ neonatal complications, severity of scorpion sting, or maternal complications. 

Scorpion stings constitute an important health concern in Iran as well as throughout the world because of their severity, extent and wide range of clinical effects. Khuzestan, in southwestern Iran, has the highest frequency of scorpionism. Despite the high prevalence of scorpionism, the effect of a scorpion sting on the mother and the fetus has not been elucidated precisely. Some animal studies have been conducted to reveal these effects [27-32]. However, their results are somewhat controversial. In addition, Kaplanoglu *et al*. [33] and Ates *et al*. [34] examined how scorpion envenomation affected pregnancy outcomes in 11 and 24 scorpion-envenomed women, respectively. The results of their study showed no significant adverse effects from scorpion stings during pregnancy on the fetus or mother. However, in the current research, 16.6% of envenomed pregnant women have adverse pregnancy outcome with preterm birth presenting the highest frequency (10.6%). These observations corroborate a study by Mendonça *et al.*, which indicated that toxin T_1_ from the venom of the Brazilian scorpion *Tityus serrulatus* induced the contraction of the isolated rat uterus due to actions on post-ganglionic autonomic nerve endings, along with acetylcholine release and stimulation of muscarinic receptors [28]. Previously, some studies had reported that venom toxins from the scorpions *Leiurus quinquestriatus* and *Pandinus exitialis* produced a powerful contraction of the rat uterus [35, 36]. Given that regular uterine contractions* *starting before 37 weeks of the pregnancy can lead to preterm labor and subsequent preterm birth [37], it can be concluded that scorpion venom can cause preterm birth, as observed in the present study. 

In another study, Ben Nasr *et al*. also concluded that scorpion envenomation may lead to abnormal uterine contraction and probably cause preterm fetal birth in pregnant women [38]. Similarly, the experimental injection of *Buthus occitanus tunetanus* venom toxin in an experimental murine model of gestation induced a dynamic dystocia during the end of pregnancy in agreement with Ben Nasr *et al*.'s study [29]. Ben Nasr *et al*. also showed that *B. o. tunetanus* crude venom induces significant increase in lipid peroxidation of maternal, placental and fetal tissues, associated with blood pressure elevation in pregnant rats; therefore, it can be life threatening perhaps leading to fetal loss and even maternal death [30]. Fetal loss in miscarriage (1.5%) and stillbirth (4.5%) also occurred in the scorpion-envenomed pregnant women of this study. However, no maternal alterations were observed.

The toxicity related to scorpion stings is, in fact, mainly due to the activity of venom toxins affecting the functioning of ion (Na^+^, K^+^, Ca^++^, Cl^-^) channels [39]. Despite the health threat posed by scorpion toxins, they are also as a potential source for human drug candidates in some cases. Scorpion venom compounds have been studied for many years as a platform for human drugs [40]. Mendonça *et al*. confirmed the cholinergic activity of toxin T_1_ of *Tityus serrulatus* that induced uterine contractions, reinforced by Neostigmine and inhibited by Atropine pregnancy [28]. On the other hand, the effect of venom toxins from the scorpions *Leiurus quinquestriatus* and *Pandinus exitialis* were indicated through release of kinins, prostaglandins and/or slow-reacting substances [35, 36]. These features of scorpion toxins might subsequently be employed to create a medication for induction of labor in post-term pregnancy. In the current study, preterm birth occurred in pregnant women envenomed by *H. lepturus*, i.e., the venom of this species also contains toxin/toxins that evoked uterine contraction and can be a candidate drug for post-term pregnancy. However, more research is necessary to prove this hypothesis. 

Pipelzadeh *et al*. reported that *H. lepturus* was responsible for 30% of the scorpion stings in Iran [41]. *A. crassicauda*is the second most dangerous scorpion in Iran [42], and was implicated by Vazirianzadeh *et al.* in 27% of scorpion stings in 2007 [43]*.*In the current study 48.5% of pregnant women were envenomed by *A. crassicauda*, and 16.7% by *H. lepturus.* There were no adverse pregnancy outcomes in pregnant women envenomed by *A. crassicauda*. However, 17.4 % and 21.9% of envenomed-pregnant women with adverse pregnancy outcomes were stung by *H. lepturus* and unknown species, respectively. There was a statistically significant relationship between scorpion species and adverse pregnancy outcomes (P-value = 0.043). The high rates of pregnancy-related complications attributed herein to *H. lepturus* envenomation may have been provoked by the powerful neurotoxic, cytotoxic and hemolytic activities of *H. lepturus* toxins [40]. These data should serve as a warning to all the inhabitants of areas with a high distribution of *H. lepturus* such as Iran and adjacent countries (Iraq, Pakistan, Saudi Arabia, Oman, Yemen and the United Arab Emirates) [12]. 

A significant relationship was found between adverse pregnancy outcomes and sting site (P-value = 0.02). Although in the present study like some other studies [34, 44, 45] the most common scorpion sting site was a lower extremity (45.5%), the stings on the trunk, head and neck (18.1%) site provoked more pregnancy-related complications compared to other sting sites. The correlation between sting site and adverse pregnancy outcomes was not clarified in the previous literature.

Among the scorpion-envenomed women whose pregnancy led to birth, all neonates following preterm birth and 6.4% of neonates following normal birth presented some prenatal-neonatal complication. Neonatal complications included Apgar score less than 8 points and birth weight lower than 2500g (Table 2). Fetal weight loss was also observed formerly in many of the viable fetuses obtained from pregnant rats treated with venom from the scorpion *A. amorexi*, the effect of which was also associated with vertebral and ossification defects in low fetal weight [46]. But herein there was no evidence of skeletal or visceral malformations in prenatal-neonatal offspring of stung women, which is consistent with the result of Dorce ALC, *et al*.'s study [32]. However, Ben Nasr *et al*. suggested that *B. o. tunetanus* scorpion envenomation during pregnancy can result in intrauterine fetal alterations and growth impairment in rats [30]. Similarly, Ismail*et al.* found that the venom of*Buthus minax*caused skeleton malformation in goats and induced fetal resorption in pregnant women stung by *B. minax* [27].

Except for four cases of low-birth-weight neonates in the present study, no complex problems were observed in relation to fetal development in scorpion-envenomed pregnant women. Nevertheless, Dorce ALC, *et al*., concluded that moderate envenomation by the scorpion*Tityus bahiensis* alters maternal reproductive performance and fetal development of pregnant female rats [32]. Overall, it seems that stings by different scorpion species may provoke different effects on neonatal outcomes.

All the results observed in the present study are a direct consequence of the action of venom toxins in pregnant women and their neonates, which should be assessed in future studies.

## Conclusion

In conclusion, scorpion envenomation can lead to pregnancy complications. There is a relationship between scorpion envenomation and adverse pregnancy outcomes. Moreover, bite location and the type of scorpion species can be significant factors for predicting an adverse pregnancy outcome in scorpion-envenomed pregnant women. It was found that *H. lepturus* can augment pregnancy complications. To investigate possible disorders, prospective studies of neonates born to scorpion-envenomed mothers, along with follow-up in the months thereafter, can provide generalizable results and additional data. 

## References

[B1] Chippaux JP, Goyffon M (2008). Epidemiology of scorpionism: a global appraisal. Acta Trop.

[B2] Furtado SS, Belmino JF, Diniz AG, Leite RS (2016). Epidemiology of scorpion envenomation in the state of Ceara, Northeastern Brazil. Rev Inst Med Trop S Paulo.

[B3] Lourenco WR (2018). The evolution and distribution of noxious species of scorpions (Arachnida: Scorpiones). J Venom Anim Toxins incl Trop Dis.

[B4] Lourenco WR (2016). Scorpion incidents, misidentification cases and possible implications for the final interpretation of results. J Venom Anim Toxins incl Trop Dis.

[B5] Sampour M (2014). Distribution of Scorpions (Arachnida: Scorpiones) of Alvar, Northpart of Andimeshk in the North of Khuzestan Province, Southwest Iran. Journal of Applied Science and Agriculture.

[B6] Kassiri H, Kassiri A, Kassiri E, Safarpor S, Lotfi M (2014). A hospital-based study on scorpionism in Khorram-Shahr County, Southwestern Iran. Asian J. Epidemiol.

[B7] Monteiro WM, de Oliveira SS, Pivoto G, Alves EC, de Almeida Goncalves Sachett J, Alexandre CN (2016). Scorpion envenoming caused by Tityus cf. silvestris evolving with severe muscle spasms in the Brazilian Amazon. Toxicon.

[B8] Sollod BL, Wilson D, Zhaxybayeva O, Gogarten JP, Drinkwater R, King GF (2005). Were arachnids the first to use combinatorial peptide libraries?. Peptides.

[B9] Lourenco WR (2018). Scorpions and life-history strategies: from evolutionary dynamics toward the scorpionism problem. J Venom Anim Toxins incl Trop Dis.

[B10] Quintero-Hernández V, Ramírez-Carreto S, Romero-Gutiérrez MT, Valdez-Velázquez LL, Becerril B, Possani LD (2015). Transcriptome analysis of scorpion species belonging to the Vaejovis genus. PLoS One.

[B11] Mohseni A, Vazirianzadeh B, Hossienzadeh M, Salehcheh M, Moradi A, Moravvej SA (2013). The roles of some scorpions, Hemiscorpius lepturus and Androctonus crassicauda, in a scorpionism focus in Ramhormorz, southwestern Iran. J Insect Sci.

[B12] Dehghani R, Kamiabi F, Mohammadi M (2018). Scorpionism by Hemiscorpius spp. in Iran: a review. J Venom Anim Toxins incl Trop Dis.

[B13] Dehghani R, Fathi B (2012). Scorpion sting in Iran: a review. Toxicon.

[B14] Amitai Y (1998). Clinical manifestations and management of scorpion envenomation. Public Health Rev.

[B15] Bahloul M, Chabchoub I, Chaari A, Chtara K, Kallel H, Dammak H (2010). Scorpion envenomation among children: clinical manifestations and outcome (analysis of 685 cases). Am J Trop Med Hyg.

[B16] Petricevich VL (2010). Scorpion venom and the inflammatory response. Mediators Inflamm.

[B17] Abdel-Rahman MA, Omran MA, Abdel-Nabi IM, Nassier OA, Schemerhorn BJ (2010). Neurotoxic and cytotoxic effects of venom from different populations of the Egyptian Scorpio maurus palmatus. Toxicon.

[B18] Undheim EAB, Fry BG, King GF (2015). Centipede venom: recent discoveries and current state of knowledge. Toxins (Basel).

[B19] Morgenstern D (2011). The tale of a resting gland: transcriptome of a replete venom gland from the scorpion Hottentotta judaicus. Toxicon.

[B20] Mosbah A, Kharrat R, Fajloun Z, Renisio JG, Blanc E, Sabatier JM (2000). A new fold in the scorpion toxin family, associated with an activity on a ryanodine-sensitive calcium channel. Proteins.

[B21] Nejati J, Saghafipour A, Mozaffari E, Keyhani A, Jesri N (2017). Scorpions and scorpionism in Iran’s central desert. Acta Trop.

[B22] Babak V, Reza HH, Banafshe A, Saeid B, Maryam MS (2010). Epidemiological study of scorpionism in the hospitals of Ahvaz, SW, Iran, 2ND six months of 2006. Jundishapur J Health Sci.

[B23] Dorce ALC, Martins AN, Dorce VAC, Nencioni ALA (2017). Perinatal effects of scorpion venoms: maternal and offspring development. J Venom Anim Toxins incl Trop Dis.

[B24] Farzanpay R (1990). A catalogue of the scorpions occurring in Iran, up to January 1986. Revue Arachonol.

[B25] Apgar V (1966). The newborn (APGAR) scoring system: reflections and advice. Pediatr Clin North Am.

[B26] Maher CG, Sherrington C, Herbert RD, Moseley AM (2003). Reliability of the PEDro scale for rating quality of randomized controlled trials. Phys Ther.

[B27] Ismail M, Ellison AC, Tilmisany AK (1983). Teratogenicity in the rat of the venom from the scorpion Androctonus amoreuxi (Aud. & Sav.). Toxicon.

[B28] Mendonca M, Da Luz MM, Freire-Maia L, Cunha-Melo JR (1995). Effect of scorpion toxin from Tityus serrulatus on the contraction of the isolated rat uterus. Toxicon.

[B29] Ben Nasr H, Hammami S, Mion G, Sahnoun Z, Chouaiekh F, Rebai T (2007). Effects of Buthus occitanus tunetanus envenomation on an experimental murine model of gestation. C R Biol.

[B30] Ben Nasr H, Serria H, Chaker S, Riadh B, Zouheir S, Kamel J (2009). Some biological effects of scorpion envenomation in late pregnant rats. Exp Toxicol Pathol.

[B31] Hmed BN, Riadh B, Serria H, Kamel J, Khaled Z (2012). Embryotoxicity following repetitive maternal exposure to scorpion venom. J Venom Anim Toxins incl Trop Dis.

[B32] Dorce ALC, Dorce VA, Nencioni ALA (2014). Mild reproductive effects of the Tityus bahiensis scorpion venom in rats. J Venom Anim Toxins incl Trop Dis.

[B33] Kaplanoglu M, Helvaci MR (2015). Scorpion stings in pregnant women: an analysis of 11 cases and review of literature. Clin Exp Obstet Gynecol.

[B34] Ates S, Karahan MA, Altay N, Akelci K, Ikiz N, Guzel B (2018). Approach to scorpion stings in pregnancy: A retrospective case series and literature review. Taiwan J Obstet Gynecol.

[B35] Osman OH, Ismail M, El-Asmar MF, Ibrahim SA (1972). Effect on the rat uterus of the venom from the scorpion Leiurus quinquestriatus. Toxicon.

[B36] Ismail M, Osman OH, Gumaa KA, Karrar MA (1974). Some pharmacological studies with scorpion (Pandinus exitialis) venom. Toxicon.

[B37] Bentley DL, Bentley JL, Watson DL, Welch RA, Martin RW, Gookin KS (1990). Relationship of uterine contractility to preterm labor. Obstet Gynecol.

[B38] Ben Nasr H, Hammami TS, Sahnoun Z, Rebai T, Bouaziz M, Kassis M (2007). Scorpion envenomation symptoms in pregnant women. J Venom Anim Toxins incl Trop Dis.

[B39] Quintero-Hernández V, Jiménez-Vargas JM, Gurrola GB, Valdivia HH, Possani LD (2013). Scorpion venom components that affect ion-channels function. Toxicon.

[B40] King GF (2011). Venoms as a platform for human drugs: translating toxins into therapeutics. Expert Opin Biol Ther.

[B41] Pipelzadeh MH, Dezfulian AR, Jalali MT, Mansouri AK (2006). In vitro and in vivo studies on some toxic effects of the venom from Hemiscorpious lepturus scorpion. Toxicon.

[B42] Pipelzadeh Jalali A, Taraz M Pourabbas R, Zaremirakabadi A (2007). An epidemiological and a clinical study on scorpionism by the Iranian scorpion Hemiscorpius lepturus. Toxicon.

[B43] Vazirianzadeh B (2005). Iranian Medical Entomology.

[B44] Mahshidfar B, Basir Ghafouri H, Yasinzadeh M, Mofidi M, Rezai M, Farsi D (2017). Demographics of scorpion sting in Iran; a cross sectional study. Emerg (Tehran).

[B45] Konca C, Tekin M, Genc Y, Turgut M (2015). Epidemiological and clinical characteristics and outcomes of scorpion envenomation in hospitalized children in Adiyaman, Turkey. Iran J Pediatr.

[B46] Ismail M, Ismail M, Ellison AC, Tilmisany AK (1983). Teratogenicity in the rat of the venom from the scorpion Androctonus amoreuxi (Aud. & Sav.). Toxicon.

